# Ultra-processed food consumption and survival in older Italians from the Moli-sani Study

**DOI:** 10.1093/eurpub/ckac129.173

**Published:** 2022-10-25

**Authors:** M Bonaccio, A Di Castelnuovo, S Costanzo, E Ruggiero, S Esposito, M Persichillo, C Cerletti, MB Donati, G de Gaetano, L Iacoviello

**Affiliations:** 1 Department of Epidemiology and Prevention, IRCCS NEUROMED, Pozzilli, Italy; 2 Cardiocentro, Clinica Mediterranea, Naples, Italy; 3 Department of Medicine and Surgery, University of Insubria, Varese, Italy

## Abstract

**Background:**

Ultra-processed food (UPF) is a major public health concern being reportedly associated with increased risk of non-communicable diseases and lower survival. However, most of the epidemiological evidence has been almost exclusively provided by research conducted in populations of youths or middle-aged adults. We tested the hypothesis that a large dietary share of UPF could be a risk factor also for vulnerable groups, as older adults (≥65 years).

**Methods:**

Longitudinal analysis on 5,215 men and women (mean age 72±5 y) from the Moli-sani Study (2005-2010, Italy) followed up for 10.9 y (median). Food intake was assessed by a 188-item FFQ. UPF was defined using the NOVA classification according to degree of processing, and categorized as quartiles of the ratio (%) between UPF (g/d) and total food consumed (g/d; weight ratio). The overall nutritional quality of the diet was measured by the Food Standard Agency nutrient profiling system dietary index (FSAm-NPS DI).

**Results:**

UPF contributed to 8% (min-max 0.0-58.4%) of the total food eaten daily and represented 14.4% (0.0-70.0%) of daily energy intake. In multivariable-adjusted analyses controlled for known risk factors, higher intake of UPF (Q4, ≥10.2% of total food), as opposed to the lowest (Q1, UPF<4.3%), was associated with increased all-cause mortality (Hazard ratio [HR]=1.19; 95%CI 1.03-1.39); these results remained unchanged after adjustment for the FSAm-NPS DI (HR = 1.21; 95%CI, 1.04-1.41). A linear dose-response relationship of 1% increment in UPF intake with all-cause mortality was also observed (p = 0.017; p for non-linearity=0.85).

**Conclusions:**

A large dietary share of UPF was associated with lower survival in older Italians consuming relatively low amounts of these foods. Expanding on previous studies on different age groups, these findings provide further justification to advise people to limit consumption of UPF even at older age.

**Key messages:**

• A large dietary share of ultra-processed food was associated with lower survival in older Italians consuming relatively low amounts of these foods.

• These findings provide further justification to advise people to limit consumption of ultra-processed food even at older age.

